# Shallow water foraminifera from Niue and Beveridge Reef (South Pacific): insights into ecological significance and ecosystem integrity

**DOI:** 10.1098/rsos.230997

**Published:** 2024-01-10

**Authors:** Shai Oron, Alan M. Friedlander, Enric Sala, Beverly N. Goodman-Tchernov

**Affiliations:** ^1^ Department of Marine Geosciences, University of Haifa, Leon Charney School of Marine Sciences, Haifa, Israel; ^2^ The Interuniversity Institute for Marine Sciences, Eilat 8828058, Israel; ^3^ Pristine Seas, National Geographic Society, Washington, DC, USA; ^4^ Hawaiʿi Institute of Marine Biology, University of Hawaiʿi, Kāneʻohe, Hawaiʿi, USA

**Keywords:** foraminifera, Niue, biodiversity, biogeography, pristine seas

## Abstract

Niue represents one of many important steppingstones facilitating the dispersal of marine organisms across the tropical Pacific Ocean. This study is part of a collaborative expedition involving National Geographic Pristine Seas, the government of Niue, Oceans 5, and the Pacific Community. We present the first survey documenting the species richness of foraminiferal communities in Niue and nearby Beveridge Reef and explore their significance for ecosystem integrity. A substantial portion (59%) of Niue's foraminiferal assemblages is Large Benthic Foraminifera (LBF), a symbiont-bearing group known as ecosystem engineers and indicators of coral reef regime shifts. LBF species reported here reflect the gradual decrease of tropical diversity from the Coral Triangle towards the central Pacific Ocean. Calcarinidae, an LBF family represented in this study by two species, is the easternmost ever recorded in published literature, and the biogeographical dispersal of this temperature-controlled group is of great importance to future global warming related studies. Foraminifera are an important component of beach development in Niue, with a close relationship between source and depositional zones. These essential ecological–sedimentary linkages highlight the importance of habitat conservation not only as a means to safeguard biodiversity, but also for its role in the island's physical framework.

## 1. Introduction

### 1.1. Significance of ecological monitoring and the role of foraminifera

Small tropical islands are highly vulnerable to natural threats and climate change-related ecological and economical risks [1–3]. Balancing conservation and sustainable development requires a proper assessment of the risks associated with pollution, overfishing, sea-level rise and changing storm intensity and patterns. To identify changes in the benthic habitat of coral reef ecosystems, simple tools applicable on a global scale along with local biodiversity baseline datasets are necessary for effective monitoring of these ecosystems. One of the best candidates for such assessments are foraminifera, which are the most diverse group of marine testate protists. Foraminifera are ideal for environmental monitoring and biogeographic studies since they show high density in marine sediments, are extremely sensitive to environmental changes, have an extensive and long researched geological record, and their shells are often preserved in the sediment after death [4–7].

The global distribution of foraminifera is controlled by both small-scale microhabitat features and large-scale oceanic patterns [7–9]. By studying current foraminiferal spatial patterns of isolated, preferably remote ‘pristine’ locations, we can uncover some of the major environmental variables controlling modern foraminifera biogeographic distributions and highlight their links to dispersal mechanisms and to the importance of habitat conservation.

Foraminifera, particularly symbiont-bearing Large Benthic Foraminifera (LBF), play a vital role in the stability and maintenance of coral reef islands, and the implications of population shifts and ecosystem change are critical to the entire ecosystem [10]. Coral reef islands are maintained through a self-sustaining mechanism relying on the deposition of calcium carbonate (CaCO_3_) by various marine organisms [10–12], with foraminifera playing a key role in this strong ecological–sedimentary linkage, where they tend to be one of the dominant sand-grade sediment sources in Pacific atolls.

### 1.2. Study area and oceanographic setting

**Figure 1.**
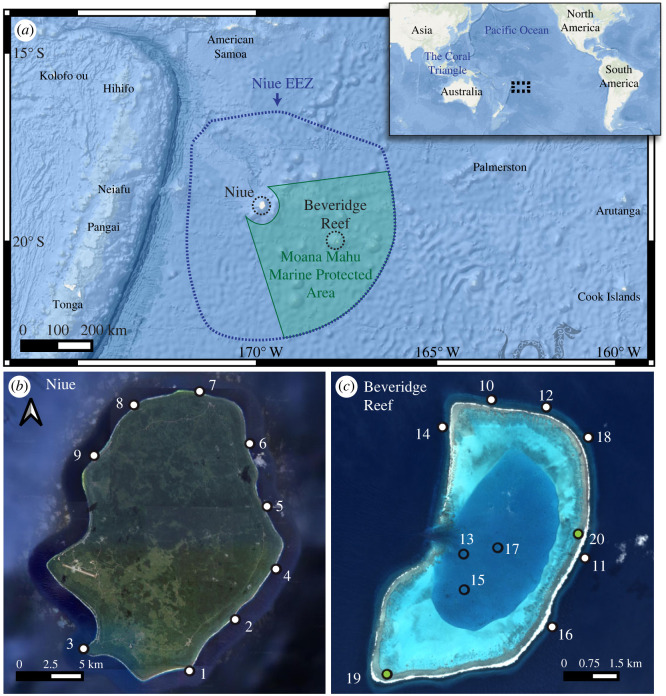
(*a*) The locations of Niue and Beveridge Reef in the South Pacific. (*b,c*) Aerial images of the islands with numbered sampling sites in the forereef (white symbols), backreef (green symbols) and lagoon (blue symbols).

Niue (figure 1) is one of the world's largest singular raised coral atolls (approx. 259 km^2^), and its Exclusive Economic Zone (EEZ) contains several seamounts and isolated coral reefs. Beveridge Reef, a semi-submerged atoll located 240 km to the southeast of Niue, is the largest of the outlying coral reefs (approx. 56 km^2^). This area is part of a series of steppingstones in the South Pacific that supports biota dispersal through the tropical Pacific Ocean. Its location between the high diversity assemblages of the Coral Triangle and the lower diversity biotas of the eastern Pacific [13,14] makes it a location of interest for biogeographic studies. Niue lies at the margin of the Indo-Pacific Warm Pool [15], and is characterized by persistent sea surface temperature of greater than 26°C. There is a distinct windward and leeward wind regime with southeast and easterly winds occurring over 60% of the time [16,17].

This study is part of a collaborative expedition between National Geographic Pristine Seas (https://www.nationalgeographic.org/society/our-programs/pristine-seas/), the government of Niue, Oceans 5 (https://www.oceans5.org/), and the Pacific Community (https://www.spc.int/). The objectives of the expedition, conducted in 2016, were to assess the biodiversity of the marine environment and contribute valuable information to the island's ongoing marine spatial planning process. Prior to this expedition, there had not been a comprehensive survey of Niue's marine ecosystems. The expedition results describe low standing stock of nearshore fishes around Niue and highlight the exceptional ecological value of Beveridge Reef as a shark refuge [18]. In October 2017 the Niue Government announced the creation of the Moana Mahu Marine Protected Area (formalized April 2020), a 127 000 km^2^ marine reserve, encompassing Niue and Beveridge Reef (https://pipap.sprep.org/pa/555705568).

While thorough foraminiferal surveys of some tropical South Pacific locations are available [19–21], previous foraminiferal taxonomic descriptions from Niue's area include only a report of several specimens from one sample collected near Alofi (the capital) during the Albatross expedition to the Pacific in 1899–1900 [22,23]. In addition, a beach development study in Niue reported proportions of the three most abundant genera in beach sediments, but not to species level [24]. Foraminifera are an extremely important component of beach development in Niue. The beaches are composed of a typical chlorozoan carbonate grain assemblage, composed of coral fragments, foraminifera and coralline algae fragments. In some locations foraminifera compose up to 81% of the sand-size beach sediment, and those sediments are suggested to be produced on the platforms close to the beaches with little longshore transport [24,25].

Here we present the first survey documenting the species richness of foraminiferal communities from shallow habitats of Niue and Beveridge Reef. The foraminiferal survey analysed at the species level improves existing knowledge of the diversity of Pacific benthic foraminiferal communities. We discuss the importance of microhabitat conservation and of general ecosystem health to preserve vital ecosystem functions related to foraminifera.

## 2. Material and methods

### 2.1. Sample collection

Sediment samples were collected in September–October 2016 during the National Geographic Pristine Seas Program campaign [18] from 15 forereef sites (11–21 m) around Niue and Beveridge Reef, two backreef sites (3 m) at Beveridge Reef and three lagoon sites (12 m) at Beveridge Reef (figure 1). Approximately 100 ml of sediment was collected by SCUBA diving from the surface of exposed patches of sediment at three randomly selected locations within each sampling site.

The characterization of benthic taxa and coral cover near the collected sediment samples involved conducting surveys along 50 m long transects parallel to the shoreline at different sampling depth strata. Benthic taxa were organized into the following functional groups: CCA (crustose coralline algae), *Lobophora* spp. (thalloid brown alga), erect algae (taxa greater than 2 cm in height except *Lobophora*), turf algae, scleractinian coral and bare substrate. A line–point intercept methodology was employed along each transect, documenting species or taxa encountered every 20 cm along the measuring tape, resulting in 250 points recorded per transect. Transects were performed at depths of 10 and 20 m at each forereef site. Additionally, at Beveridge Reef, five more transects were conducted in the backreef area, absent around Niue, at an approximate water depth of 1 m. The sandy lagoon sites at Beveridge Reef were classified as bare substrate, specifically termed as ‘lagoon sand’. Data collection methods and results are described in detail in Niue's Pristine Seas expedition scientific report [18].

The Government of Niue, Ministry of Natural Resources granted all necessary permits to conduct this research. No vertebrate or cephalopod sampling was conducted and therefore no approval was required by any animal care and use committee.

### 2.2. Sample analysis, taxonomy, and imaging

The analysis methods described here are a replication of the methods used in a previous publication resulting from the same collaborative project with the National Geographic Society [26]. Sediment samples were preserved immediately after each dive in 95% ethanol with Rose Bengal solution (2 g l^−1^) for at least four weeks. Before analyses, one sample per site was drained, gently mixed with a glass rod to avoid grain-size sorting, and a subsample of 10 ml was taken by removing sediment with a spatula into a 15 ml Eppendorf® tube up to the 10 ml mark. The two remaining samples were archived. Subsamples were wet sieved on 63 and 125 µm mesh size sieves, and then dried. Each fraction was divided with a microsplitter, reaching 1/8 splits. Each split used was dry picked fully, until at least approximately 250 specimens were picked overall (all specimens greater than 63 µm) [6,27]. If the approximately 250 specimens target was not achieved after picking the entire 10 ml subsample (all splits), another 5 or 10 ml subsample was processed. If a 20 ml subsample did not achieve the target, only then was a value smaller than 250 individuals included in the calculations. The specimens were sorted by species in separate cell slides for each sampling station, fixed with glue, documented, and counted. Stained specimens, with Rose Bengal coloration visible inside the shell, were considered living at the time of sampling. Grainsize distribution was analysed using the remaining sample residues (approx. 80–90 ml). The sediment was dried, weighed on an analytical scale, wet sieved with sieves of mesh sizes 63, 125, 250, 500 and 1000 µm, and then dried again. Each fraction was weighed separately. The grainsize percentages were calculated in reference to the total dry weights.

Species were identified using an online database [28], classic taxonomic literature [22,23,29–32], and reported literature of assemblages from other Pacific locations that included images or illustrations [20,21]. In the relevant literature, species were occasionally reported with various different genus or species names. We checked for synonymy, as updated for January 2023, using the WoRMS Taxon match tool (https://www.marinespecies.org/aphia.php?p=match), validating species accepted names, authorities, and classifications.

Light images were taken with an Olympus EPL-1 digital camera fitted on a Nikon SMZ645 stereo microscope, and with a Nikon DS-Fi2 camera fitted on a Nikon Eclipse ci microscope at the Interuniversity Institute for Marine Sciences (IUI), Eilat, Israel. Scanning electron microscope (SEM) image acquisition was performed on a Zeiss Sigma HD high-definition field emission SEM at the Bioimaging Unit, Faculty of Natural Sciences, University of Haifa, Israel. Satellite image based maps were created using the free and open source QGIS [33].

### 2.3. Statistical analysis

Statistical analysis was based on dead assemblages, reflecting the long-term contribution of generations of living fauna that formed the sediment. Live (stained) foraminifera data are given in the electronic supplementary material but do not represent the actual standing stock in the studied habitats since the sampling was from sediment patches only, and not from algae mats or hard substrate.

Canonical correspondence analysis was used to explore factors defining the different habitats (benthic functional groups) and conceivably effecting distribution of key species of foraminifera with greater than 1% abundance. The benthic functional groups applied were: CCA, *Lobophora* (thalloid brown algae), erect algae (taxa greater than 2 cm height except *Lobophora*), turf algae, coral, and bare substrate, measured in proximity to each sampling location [18]. Fisher's alpha diversity index was calculated to define the relationship of the number of species to the number of individuals in the assemblages. All statistical analysis was computed using PAST software v.4.03 [34].

## 3. Results

**Plate 1.**
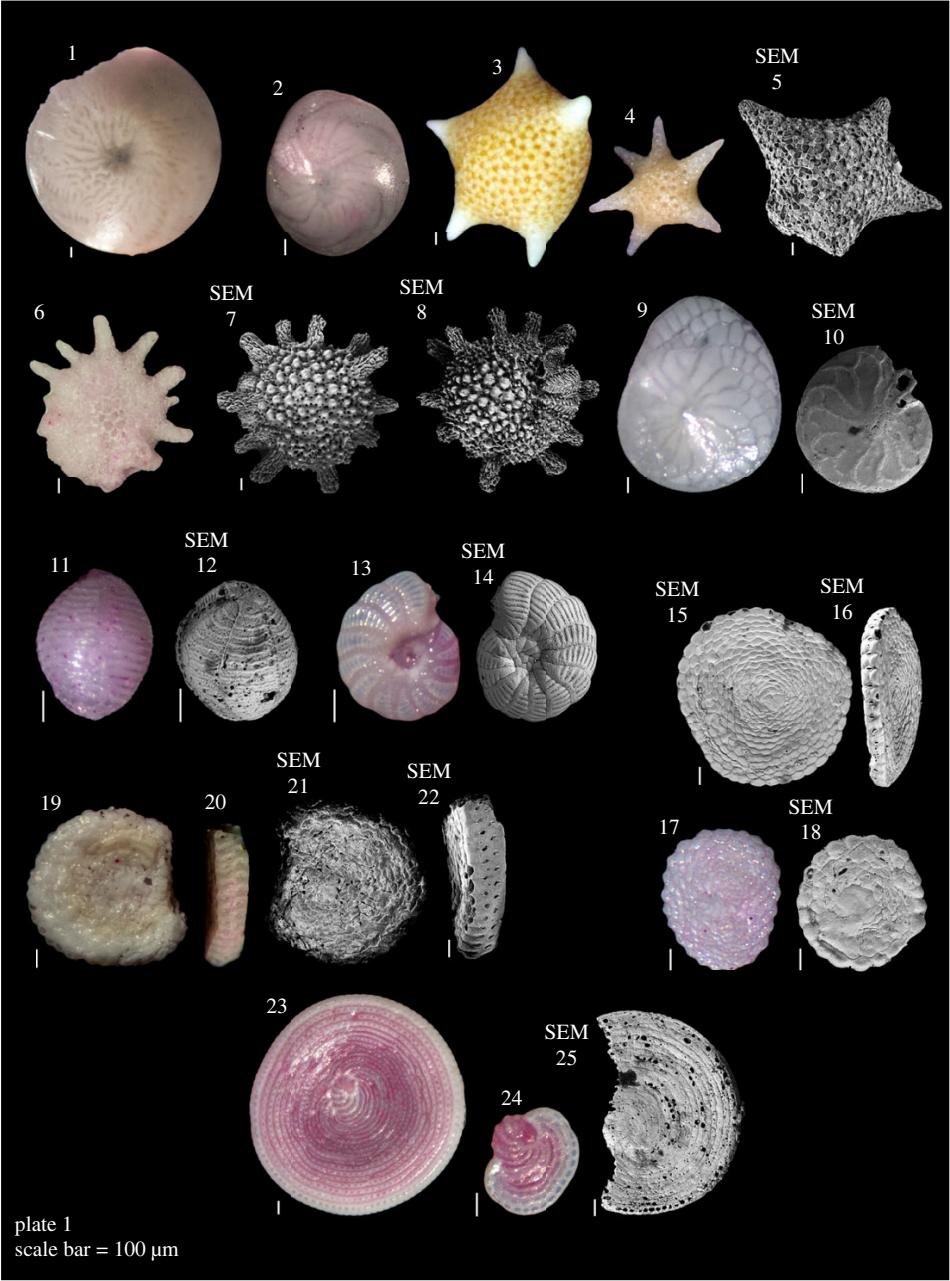
**LBF: 1** *Amphistegina lobifera* Larsen, 1976; **2** *Amphistegina lessonii* d'Orbigny in Guérin-Méneville, 1832; **3–5** *Baculogypsina sphaerulata* (Parker & Jones, 1860); **6–8** *Calcarina hispida* Brady, 1876; **9,10** *Heterostegina depressa* d'Orbigny, 1826; **11,12** *Borelis schlumbergeri* (Reichel, 1937); **13,14** *Peneroplis pertusus* (Forsskål in Niebuhr, 1775); **15–18** *Sorites orbiculus* (Forsskål in Niebuhr, 1775); **19–22** *Amphisorus hemprichii* Ehrenberg, 1839; **23–25** *Parasorites orbitolitoides* (Hofker, 1930).

**Plate 2.**
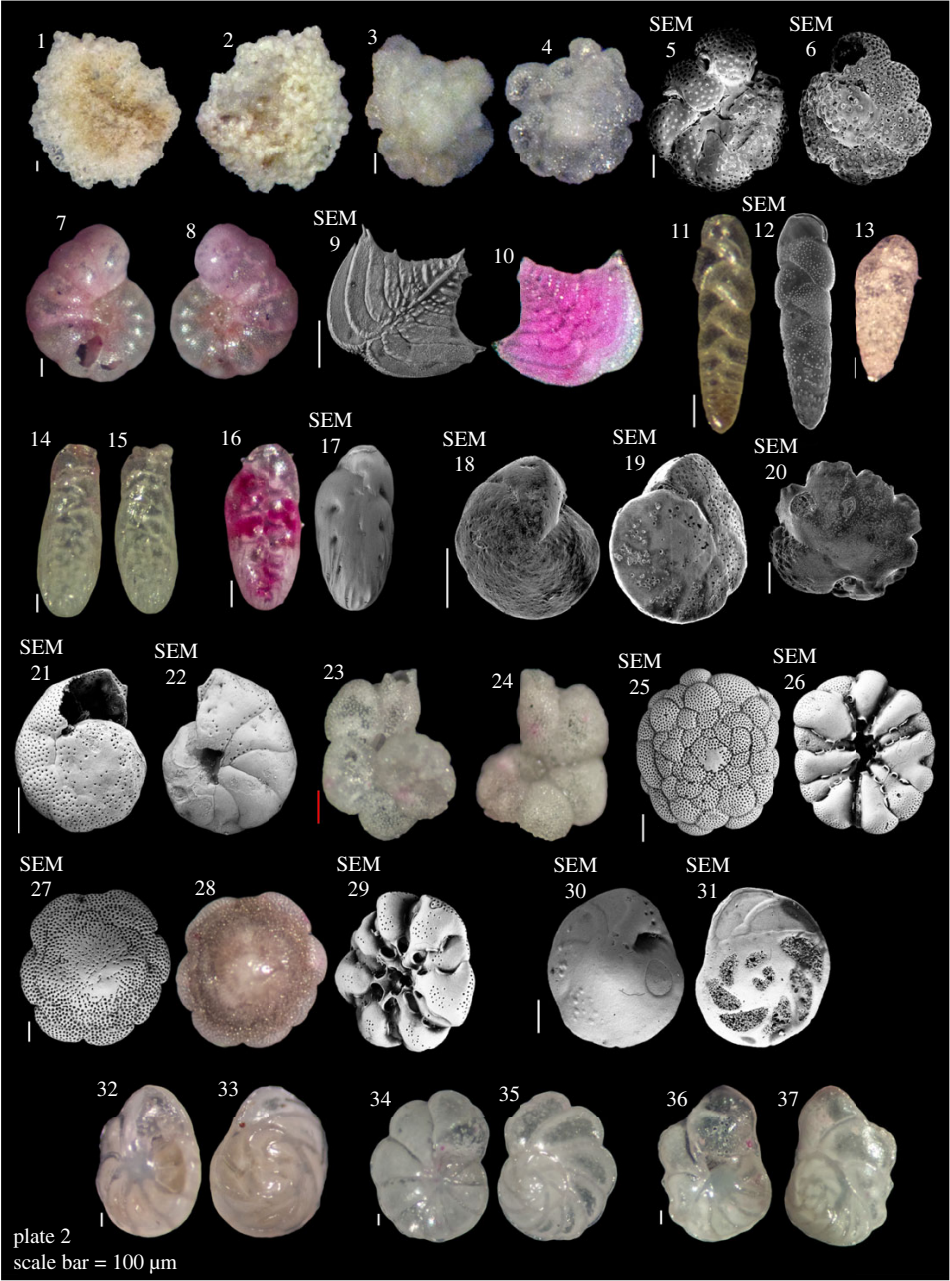
**1,2** *Planogypsina acervalis* (Brady, 1884); **3–6** *Acervulina mabahethi* (Said, 1949); **7,8** *Anomalinoides* sp.; **9,10** *Rugobolivinella elegans* (Parr, 1932); **11,12** *Neocassidulina abbreviate* (Heron-Allen & Earland, 1924); **13** *Bolivina variabilis* (Williamson, 1858); **14–17** *Loxostomina* sp.; **18,19** *Cibicides mabahethi* Said, 1949; **21,22** *Cibicides phillipensis*? Collins, 1974; **23,24** *Lobatula lobatula* (Walker & Jacob, 1798); **25,26** *Cymbaloporetta bradyi* (Cushman, 1915); **27–29** *Cymbaloporetta squamosa* (d'Orbigny, 1839); **20** *Asanonella tubulifera* (Heron-Allen & Earland, 1915); **30–33** *Eponides repandus* (Fichtel & Moll, 1798); **34,35** *Eponides repandus*? (Fichtel & Moll, 1798); **36,37** *Poroeponides lateralis* (Terquem, 1878). The red scale bar for **23**,**24** is an estimate.

**Plate 3.**
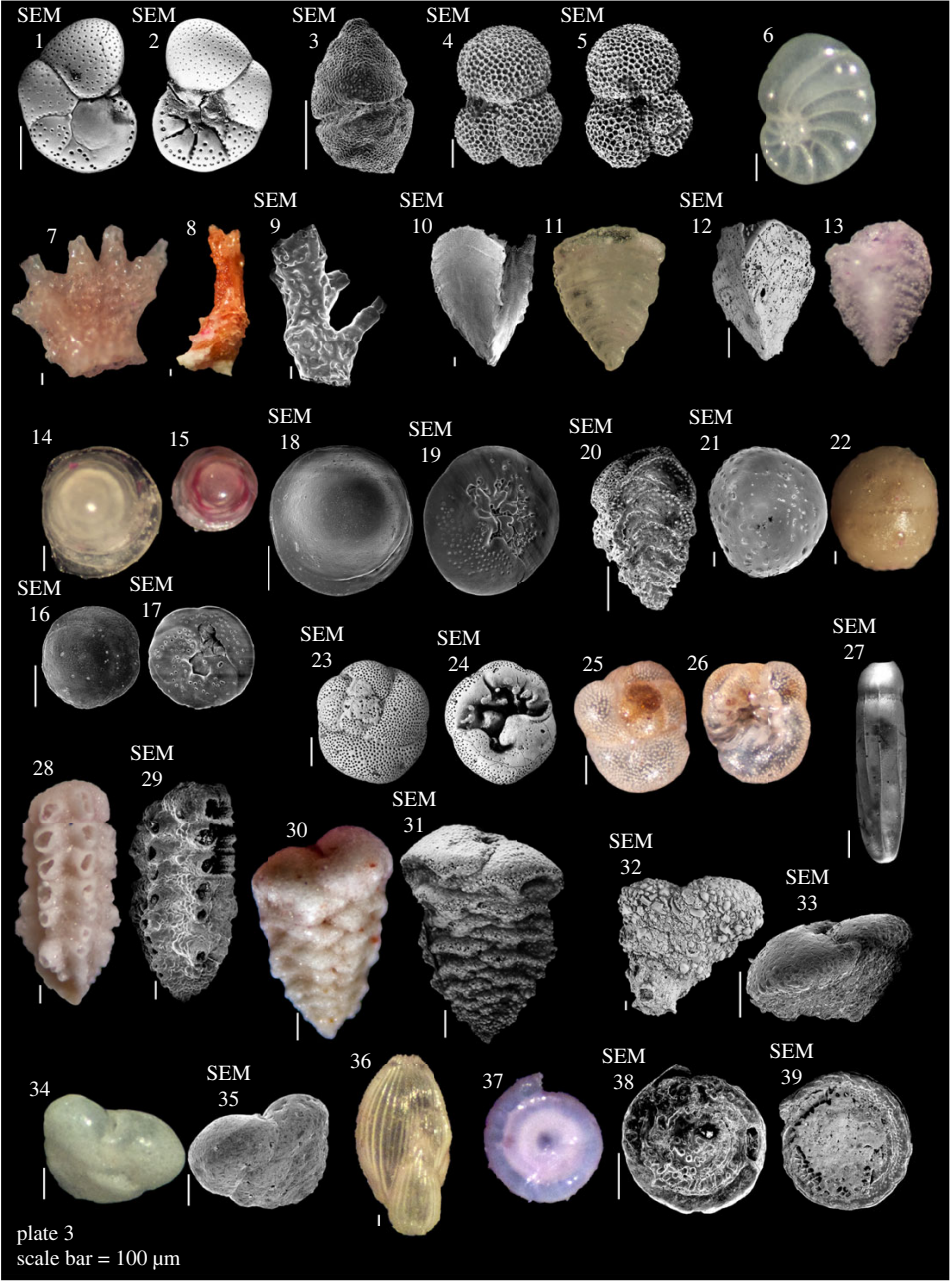
**1,2** *Anomalinulla* sp.; **3** *Glabratellina kermadecensis* Hayward, Grenfell, Reid & Hayward, 1999; **4,5** *Globigerinoides ruber* (d'Orbigny, 1839); **6** *Nonionoides* sp.; **7–9** *Homotrema rubrum* (Lamarck, 1816); **21,22** *Kuremsia papillate* (Heron-Allen & Earland, 1928); **10,11** *Chrysalidinella fijiensis* Cushman, 1945; **12,13** *Fijiella simplex* (Cushman, 1929); **20** *Reussella spinulosa* (Reuss, 1850); **14–19** *Neoconorbina tuberocapitata* (Chapman, 1900); **23–26** *Rosalina orientalis* (Cushman, 1925); **27** *Siphogenerina raphanus* (Parker & Jones, 1865); **28,29** *Siphoniferoides siphonifer* (Brady, 1881); **30,31** *Sahulia kerimbaensis* (Said, 1949); **32** *Sahulia lutzei* Langer, 1992; **33** *Sahulia conica*? (d'Orbigny, 1839); **34,35** *Textularia semialata*? Cushman, 1913; **36** *Sigmomorphina*? sp.; **37** *Cornuspira*? sp.; **38,39** *Cornuspira* sp. 1.

**Plate 4.**
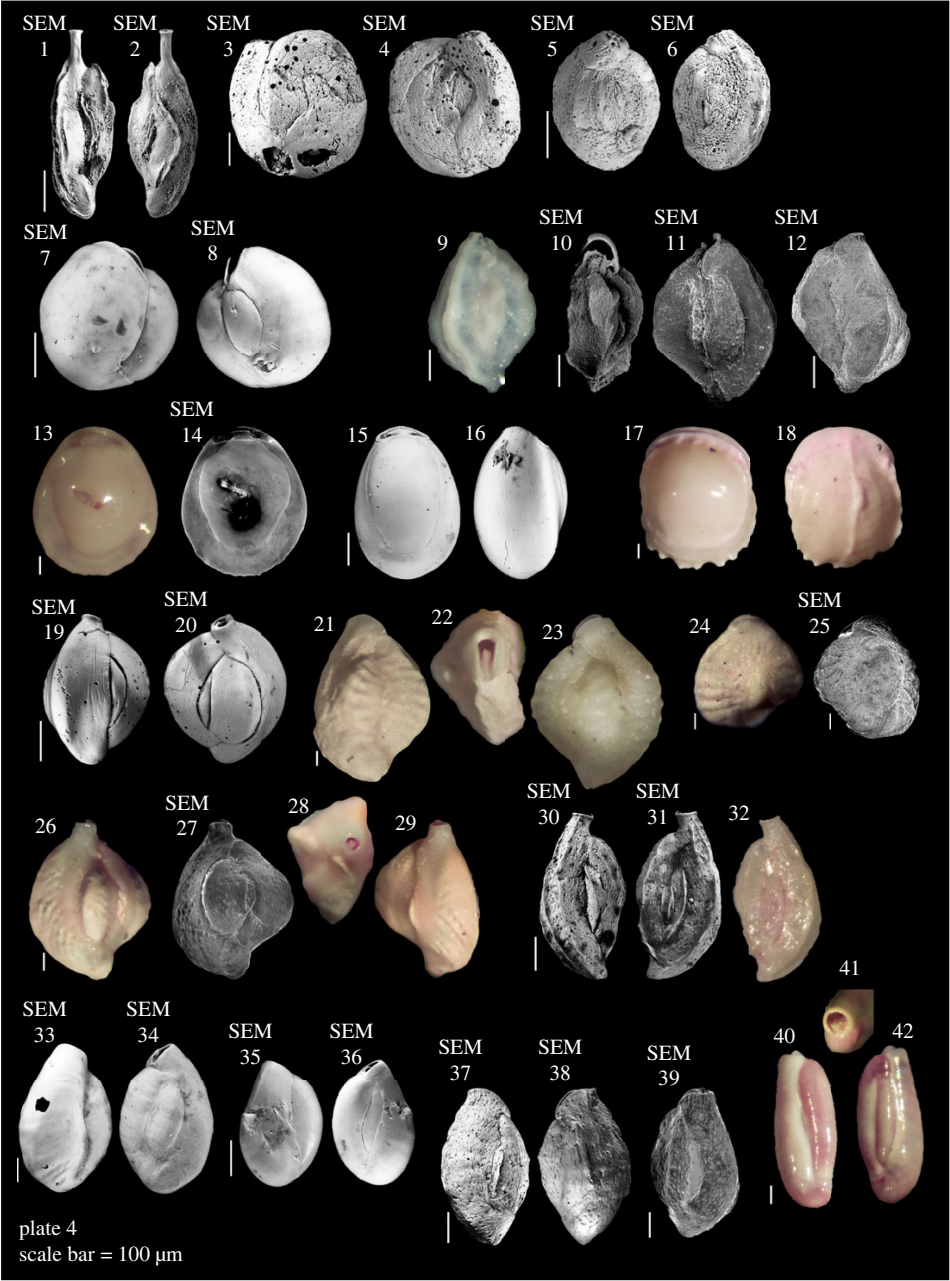
**1,2** *Sigmoilinella* cf. *S. tortuosa* Zheng, 1979; **3–6** *Hauerina pacifica* Cushman, 1917; **7,8** *Miliolinella circularis* (Bornemann, 1855); **9–12** *Miliolinella oceanica* (Cushman, 1932); **13,14** *Pyrgo* sp. 1; **15,16** *Pyrgo* sp. 2; **17,18** *Pyrgo denticulate* (Brady, 1884); **19,20** *Quinqueloculina* cf. *Q. cuvieriana* d'Orbigny, 1839; **21–23** *Quinqueloculina parkeri* (Brady, 1881); **24,25** *Quinqueloculina parkeri*? (Brady, 1881); **26–29** *Quinqueloculina philippinensis*? Cushman, 1921; **30–32** *Quinqueloculina polygona*? d'Orbigny, 1839; **33–36** *Quinqueloculina seminulum*? (Linnaeus, 1758); **37–39** *Quinqueloculina* sp. 1; **40–42** *Quinqueloculina* sp. 2.

**Plate 5.**
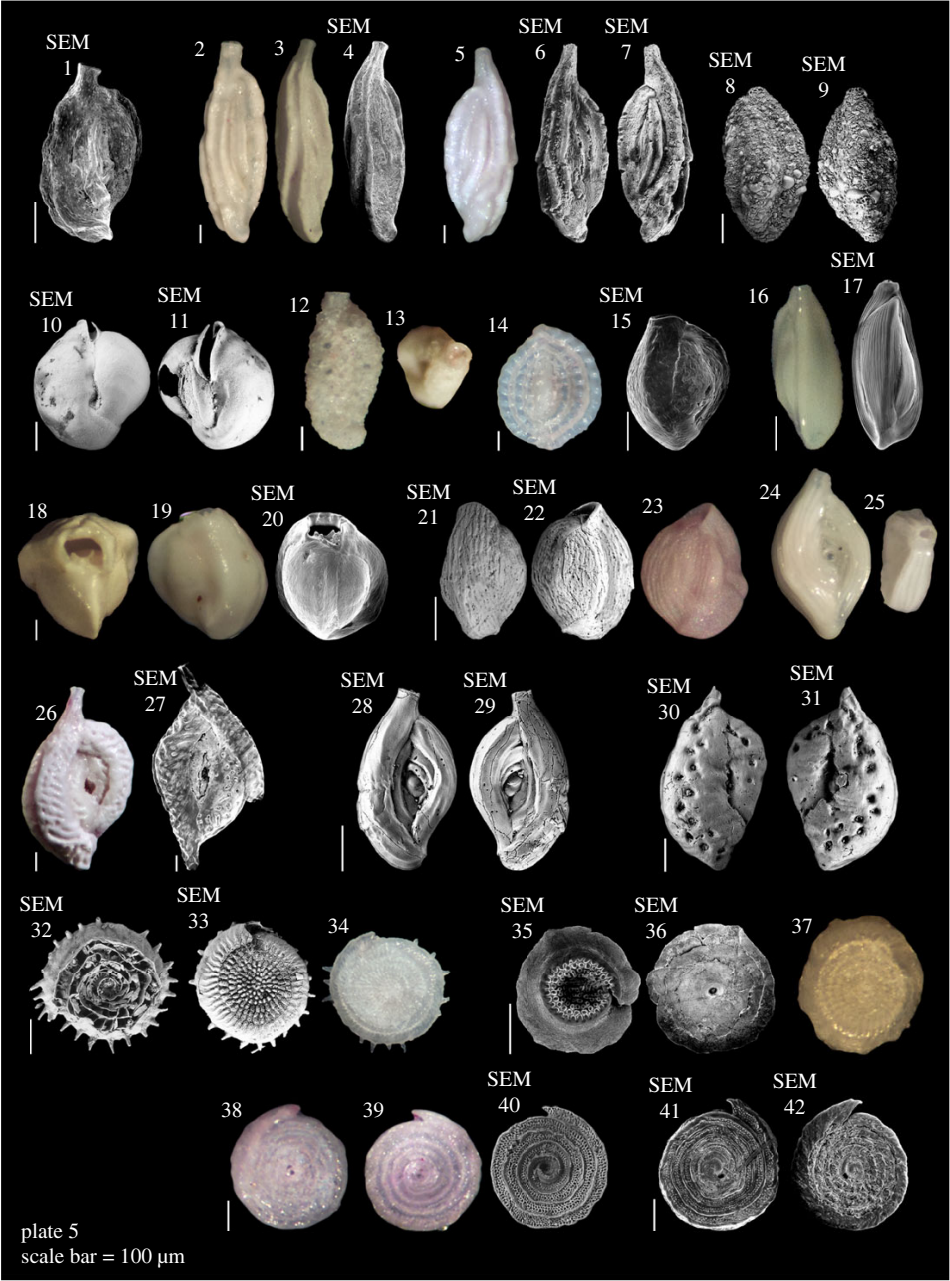
**1** *Quinqueloculina zhengi* Parker, 2009; **2–7** *Quinqueloculina sulcate* d'Orbigny in Fornasini, 1900; **8,9** *Schlumbergerina alveoliniformis* (Brady, 1879); **10,11** *Sigmamiliolinella australis*? (Parr, 1932); **12,13** *Siphonaperta* sp.; **14** *Spirosigmoilina bradyi* Collins, 1958; **15** *Triloculina* sp. 1; **16,17** *Triloculina* sp. 2; **18–20** *Triloculina* sp. 3; **21–23** *Varidentella neostriatula* (Thalmann, 1950); **24,25** *Spiroloculina corrugate*? Cushman & Todd, 1944; **26,27** *Spiroloculina* sp.; **28,29** *Spiroloculina antillarum* d'Orbigny, 1839; **30,31** *Spiroloculina foveolate* Egger, 1893; **32–34** *Conicospirillinoides* cf C. *elaborates* Cheng & Zheng, 1978; **35,36** *Conicospirillinoides* sp.; **37** *Conicospirillinoides*? sp. **38–42** *Planispirillina tuberculatolimbata* (Chapman, 1900).

Most samples in this study were dominated by medium to coarse sand, with an average of 91% of sediment grains greater than 250 µm (electronic supplementary material, table S1; table 1). The sediments were composed of a typical chlorozoan assemblage, e.g. coral fragments, foraminifera, CCA, *Halimeda*, molluscs, and echinoid spines. We identified 76 species of foraminifera belonging to 52 genera (electronic supplementary material, table S2; plates 1–5). The assemblages consisted of mostly epiphytic taxa and taxa associated with coral reefs, hard substrate and coarse grain habitats. Rotaliida was the most abundant order in forereef sites in Niue (90 ± 11%) and Beveridge Reef (72 ± 13%) and decreasing in Beveridge Reef's backreef (53 ± 10%). Miliolida was the most abundant order in Beveridge Reef's lagoon (89 ± 15%) (table 1). *Amphistegina lobifera* was the most dominant species, accounting for 35% of all individuals counted, followed by *Homotrema rubrum* (15%), *Calcarina hispida* (7%) and *Sorites orbiculus* (5%) (electronic supplementary material, table S2). Symbiont-bearing LBFs were a major component accounting for 59% of the assemblages, represented by six families: Amphisteginidae, Calcarinidae, Nummulitidae, Alveolinidae, Peneroplidae and Soritidae. Dead assemblage Fisher's alpha diversity index ranged between 0.6 and 12.2 in individual samples. Live individuals were found to only be 3% of the total individuals counted (electronic supplementary material, table S2) and were excluded from the statistical analysis. The assemblages may include a number of yet unreported species, given here in open nomenclature (see plates 1–5).

**Table 1.** Group proportions summary (average ± s.d.) and Fisher's alpha diversity index of foraminifera based on counts presented in electronic supplementary material, tables S1 and S2.

	Niue forereef	Beveridge forereef	Beveridge backreef	Beveridge lagoon	total
water depth (m)	11–21	14–20	3	12	
Fisher's alpha	4.7 ± 3.7	5.4 ± 3.1	3.3 ± 1.2	3.1 ± 3.4	4.5 ± 3.2
% LBF	73 ± 13	46 ± 29	47 ± 5	48 ± 32	59 ± 24
% Rotaliida	90 ± 11	72 ± 13	53 ± 10	10 ± 15	69 ± 30
% Miliolida	7 ± 10	17 ± 11	41 ± 9	89 ± 15	26 ± 31
% Textulariidae	2 ± 1	7 ± 8	3 ± 4	1 ± 1	3 ± 5
**grain size**					
% >1000 µm	32 ± 29	34 ± 29	19 ± 14	8 ± 6	28 ± 26
% 500–1000 µm	41 ± 22	33 ± 17	35 ± 10	34 ± 21	37 ± 19
% 250–500 µm	23 ± 18	23 ± 18	40 ± 17	32 ± 7	26 ± 17
% 125–250 µm	3 ± 6	8 ± 16	6 ± 6	15 ± 16	7 ± 11
% 63–125 µm	0	1 ± 2	0	9 ± 15	2 ± 6
% <63 µm	0	1 ± 1	0	2 ± 2	1 ± 1

**Figure 2.**
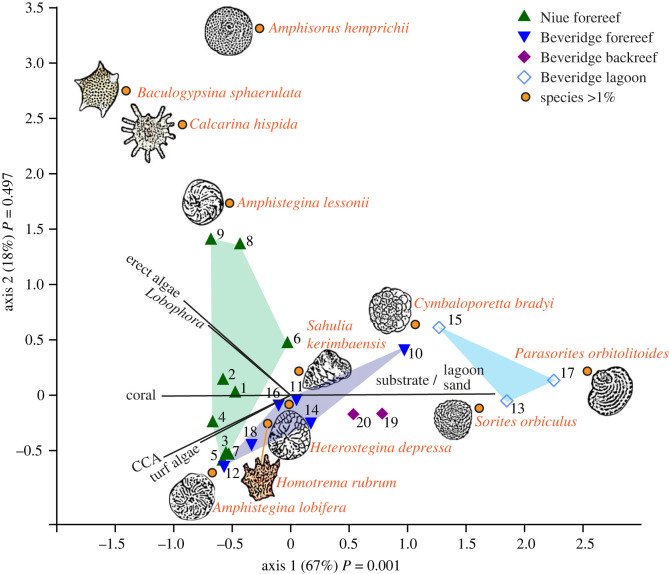
Canonical correspondence analysis biplot of foraminifera with greater than 1% abundance and benthic functional groups.

**Figure 3.**
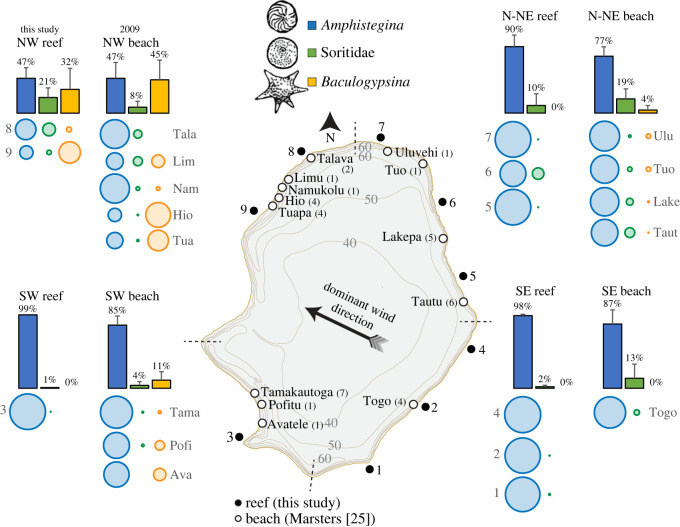
Average proportions of three key Large Benthic Foraminifera (LBF) taxa previously analysed in 2009 from beach sediments in Niue [25] and from reef sediments collected during this study. The outlined map of Niue island denotes the locations of reef and beach sampling stations. Coloured bars illustrate the averages for each section (NW, N-NE, SE, SW), while coloured circles represent the proportions at individual sampling stations. Numbers in parentheses following beach names indicate the number of duplicates. Error bars, demonstrating standard deviation (SD), are included. For more detailed data, refer to electronic supplementary material, table S4.

Canonical correspondence analysis for foraminifera with greater than 1% abundance and benthic habitat factors showed that the different sites (Niue forereef and Beveridge forereef, backreef and lagoon) were mostly well separated in ordination space. Erect algae and reef building corals explained much of the variation for Niue, while bare substrate (including lagoon sand) was responsible for explaining much of the benthic composition at Beveridge Reef. Wind protected, erect algae-rich sites were mostly correlated with the presence of Calcarinidae (*Baculogypsina sphaerulata* and *C. hispida*) and *Amphisorus hemprichii.* Backreef and lagoon sites were home mostly to the delicate representatives of the miliolid LBFs, *Parasorites orbitolitoides* and *Sorites orbiculus* (figure 2; electronic supplementary material, table S3). Proportions of three key LBF taxa in reef sediments sampled during this study around Niue showed some similarity to proportions of those LBF taxa previously analysed in beach sediments around Niue [25] (figure 3; electronic supplementary material, table S4).

## 4. Discussion

### 4.1. Biodiversity

This survey provides the first illustrated foraminiferal catalogue from shallow reef and near shore habitats in Niue's EEZ. The dead assemblages only are discussed here as they reflect the long-term contribution of generations of living fauna that formed the sediment composition over the course of many foraminiferal life cycles, averaging transport mechanisms from the surrounding coral reef and algae mats, taphonomy processes, and seasonal effects [5,35,36]. Also, because these samples are from sediment patches rather than this foraminifera community's preferred living habitats (the surrounding algal mats or hard substrates), the proportion of living (stained) tests was predictably low (electronic supplementary material, table S2). Nevertheless, the live count data might become useful for future monitoring since even though live symbiont-bearing LBFs generally are much less abundant in sediments than on algal mats or hard substrate, a case study from Kirimati Island showed that live LBFs were significantly more common in sediments collected at less impacted sites than near human impacted sites [37].

Overall, the composition of the assemblages analysed here shifted from Rotaliid (hyaline wall structure) dominated in forereef sites in Niue (90 ± 11% Rotaliida) and Beveridge Reef (72 ± 13% Rotaliida) to intermediate prevalence in Beveridge Reef's backreef (53 ± 10% Rotaliida), while Miliolid (porcelaneous wall structure) dominated in Beveridge's lagoon (89 ± 15% Miliolida). This is a typical community structure that corresponds with the known distribution of modern reef-associated foraminifera [38,39]. This distribution correlates to physico-chemical condition changes along the atoll habitats because shallow-water miliolinds are generally able to tolerate higher salinities and temperatures than rotaliides, and therefore are expected to be more common in the shallower and more evaporative back lagoon [40–42].

The foraminiferal greater than 63 µm assemblages consist of 59% LBF species, on average, with some samples showing up to 89%, and up to 85 LBF individuals per millilitre of sediment (electronic supplementary material, table S1). As such, the majority of species and of foraminiferal-produced sediment volume is attributed to LBFs. The distribution of these species is controlled by light intensity, temperature, water energy, substrate type, nutrients and water quality [8,43–46]. LBFs, like corals, host eukaryotic symbionts as well as a complex prokaryotic microbiome [47–50]. They require similar conditions to reef-building corals to thrive and show high sensitivity to physico-chemical conditions, so they are ideal for assessing the impact of environmental change in tropical and sub-tropical areas and can be good proxies for ecological regime shifts in coral reef environments [50–53]. Likewise, shifts in assemblages of the symbiont bearing LBFs may detect early signs of degradation in the reef benthic habitat [46,54]. Thus, detecting ecological regime shifts might be possible by annually surveying the living assemblage of LBF or surveying the dead assemblage every few years [53] to identify trends before major changes occur.

The most abundant family in this study, Amphisteginidae (38% of total counts), is a diatom-bearing LBF that occurred largely throughout the sampling stations but was found to have its highest concentrations within Niue samples, where the habitat was defined by higher concentrations of algae relative to Beveridge. Also, the next most abundant members of the LBF family Calcarinidae (*Baculogypsina sphaerulat* and *C. hispida*) were found in high numbers mostly in sampling stations off the west and northwest shores of Niue (figure 2). The topographic relief of Niue island (an uplifted atoll) provides a more sheltered leeward shore relative to Beveridge Reef, which is emergent only at low tide. This protection likely provides a more stable environment for the growth of erect algae and corals [18], supporting Calcarinidae community. Additionally, Beveridge Reef is also more exposed to storms, and it has been impacted by a number of cyclones in recent years, possibly affecting coral size distribution and the growth of erect algae [18]. Despite its high exposure, Beveridge Reef is characterized by higher habitat diversity than Niue because it also contains backreef and lagoon sites, not present in Niue. This is represented at Beveridge by the presence of two miliolid LBFs, *Sorites orbiculus* and *Parasorites orbitolitoides*. Those known low-energy settings/lagoon dwellers were found mostly in Beveridge Reef's lagoon, while their epifaunal/epiphytic relative *Amphisorus hemprichii* was found mostly in Niue defining the wind and swell protected erect algae habitats.

Besides LBFs, species that composed more than 1% of the assemblages were *Cymbaloporetta bradyi* (Rotaliida defining mostly station 10 in Beveridge Reef), *Sahulia kerimbaensis* (Textulariida), and *Homotrema rubrum*, all relatively large in size and contributing significantly to sedimentation. *Homotrema rubrum*, which was very abundant throughout the forereef and backreef sites in both islands, is a unique benthic tropical and sub-tropical red sessile epiphytic foraminifera that reinforces the coral-reef framework by calcifying in cracks, crevices, and on the cryptic undersides of coral colonies and other reef substrata [55,56]. The origin of its red pigment is not definitive [57], and it is a very important sediment component in some locations—for example it is responsible for the famous pink sands in Bermuda [58]. Its ease of identification and changing pigment intensity when exposed has led to studies using it as a proxy for storm and tsunami deposit interpretation [59]. *H. rubrum* was previously recommended as a model species for examining natural environmental variability and anthropogenic impacts [57,60]. For all these reasons, *H. rubrum* is a model species for conducting benthic ecological surveys.

### 4.2. Biogeography

Biogeographically, Niue is located well to the east of the Coral Triangle (Indonesia, Malaysia, the Philippines, Papua New Guinea, Timor Leste and the Solomon Islands) and represents an important link between the Coral Triangle, considered to be the centre of global marine biodiversity [61–64], and other central and eastern Pacific islands. LBF assemblages recorded in this study show some similarities to assemblages recorded in other parts of the Pacific [8,19,45,65–70], and LBF diversity (10 species) reflects the gradual decrease of coral reef diversity towards the central Pacific Ocean [13,71,72]. Hence, occupying a transitional position between the high diversity Coral Triangle (greater than 39 species of LBFs) and the low diversity areas of the tropical eastern Pacific where less than 6 species of LBFs were recorded in the Galapagos [73], and in the Revillagigedo Archipelago and Clipperton Atoll (own unpublished data). Decreasing LBF species richness towards the tropical eastern Pacific closely matches diversity patterns observed in scleractinian reef corals and other invertebrates [13,14,61,74], indicating a strong biogeographic barrier of westward flowing currents that obstructs eastward directed dispersal.

The Calcarinidae foraminifera family, represented in this study by *C. hispida* and *B. sphaerulata*, are an intriguing biogeographical component in Niue. They were completely absent from the Beveridge Reef samples but *C. hispida* was found in almost all Niue samples. The Calcarinidae are exclusive to the Indo-Pacific and are extremely abundant on reef crests in the western tropical Pacific. With the exception of the small *Neorotalia calcar*, calcarinids exhibit a relatively restricted distribution possibly because of their inability to tolerate lower temperatures [8,13,75]. Species distribution models have shown that *Calcarina* spp. prefer areas with annual mean temperatures of ≥ 28°C, and their occurrence within the Indo-Pacific region appears to correspond to the 24°C isotherm [75]. Additionally, calcarinids are known to thrive in reef habitats dominated by algae, and this association could be an important aspect for future monitoring and distribution modelling [76]. The calcarinids in Niue are the easternmost ever recorded, first documented in Niue's beach development study [24] and now in this study. Prior to this, Samoa was the known easterly dispersal limit [8,77]. The knowledge across the east Pacific is patchy but the current understanding is that calcarinids are absent from the Tuamotu Archipelago [19], Moorea [21] and Kiritimati (Christmas) Island [37], all approximately 2000–2500 km to the east or northeast of Niue. They were also reported as absent from Rarotonga, Cook Islands [78] which lies approximately 1000 km to the east of Niue. Any future reporting of their presence further east or northeast could be a useful indicator of the expansion of the Indo-Pacific Warm Pool conditions suitable for their habitat. Hence, updated biogeographical data on this temperature controlled group will contribute to predicted marine distribution ranges in the face of global warming [75].

### 4.3. Importance to beach development

On a local scale, the sediments in Niue and Beveridge Reef are dominated by medium to coarse sands (table 1), with very limited deposition of silt, clay, and fine sand as a result of the distance from continental influence. This habitat is defined as a ‘carbonate environment’, receiving little to no clastic material from terrestrial erosion. In carbonate environments the biogenic carbonate provides the vast majority of the sediment supply because it is not diluted nor supplemented by clastic material transported to the coastline either by rivers or runoff [5]. Therefore, an essential ecological–sedimentary linkage exists, as almost all sediment is derived from reefs or reef-related biota; and therefore the ramifications of any changes in the nearshore and shallow marine ecosystem stability can have profound geomorphological consequences for the coastlines of coral reef islands [10,79].

Foraminifera are known as an essential component in these reef island sediment budgets and dynamics [10,12,67,80–82]. In some Indo-Pacific coral reefs, foraminifera calcium carbonate production can contribute approximately 30–90% of the reef and nearshore deposits [67,83,84]. In carbonate beach sands foraminifera can reach ratios of up to 95% [80,85], and mass culturing of the LBF *B. sphaerulata* (star sands) was even examined as means to protect island coasts against sea-level rise [86]. Foraminifera concentrations are usually elevated in beach sediments compared to reef deposits since they have a short community turnover and are easily transported and dispersed immediately following death [83]. Sediments of coral origin generally require an erosion phase from the reef framework followed by further reductions in sediment size before they are of a suitable size for transport to the beach [10,87]. This is in agreement with the composition of Niue's shores where foraminifera compose, on average, 19% of the sand-size beach sediment, reaching values of up to 81% foraminifera in the northwestern beaches [24,25].

Diverse dead assemblages with large variability between locations and distinct species-location preferences (figure 2), as well as relatively similar proportions of key species in the reef and beach assemblages—with *Baculogypsina* found almost exclusively in beaches that are close to wind-protected reef habitats (figure 3)—support previous geomorphological research suggesting that Niue's sediments are produced on the platforms close to the beaches with little evident longshore transport [24,25]. The close relationship between source and depositional zones is a further indication that the beaches are highly vulnerable to any change in either energy conditions or ecological disturbance affecting this sediment supply.

If empty test transport and deposition are disrupted by habitat destruction or by the diversion of near shore currents (for example, by construction of seawalls), test accumulation and abundances in beach deposits may change drastically even if *in situ* production remains unchanged [80,82]. There are many valuable lessons for coastal zone management related to disrupted beach sediment regimes [88]. An unfortunate case study for interrupting the connection between sand producers and the deposition area was documented in Candi Dasa, Bali (Indonesia), where deepening of a moat for tourist beaches completely eroded beach sand by increased wave-induced currents in combination with storm events [89]. Even overfishing can result in loss of beaches due to changes in trophic structure and nutrient cycling, in turn affecting foraminifera populations and thus sediment supply [37,54]. Sediments in Niue are fundamentally linked to the reef biota and their dynamics is also dependent on the frequency of cyclones removing sand from the beaches. Fast recovery of the foraminifera-rich northwestern beaches was previously observed after Tropical Cyclone Heta (2004), suggesting that the foraminifera community can re-establish sediment budget faster after cyclones compared to other calcifying groups [25]. This highlights the importance of microhabitat conservation, general ecosystem health, and water quality to the structural integrity of the beaches and the reef framework.

## 5. Conclusion

This study provides shallow water foraminiferal ecological baseline information from the newly established marine protected area of Niue and Beveridge Reef. Symbiont bearing LBFs and the sessile red pigmented species *H. rubrum* dominate the assemblages in Niue and Beveridge Reef and should be monitored closely as shifts in their communities may detect early signs of degradation in the reef benthic habitat. LBFs in this study are represented by 10 species belonging to six families: Amphisteginidae, Calcarinidae, Nummulitidae, Alveolinidae, Peneroplidae and Soritidae. This species composition represents a transitional position between the high diversity Coral Triangle and the low diversity areas of the tropical eastern Pacific. Calcarinidae, represented in this study by *C. hispida* and *B. sphaerulata*, are the eastmost ever recorded in published literature, and the biogeographical dispersal of this temperature-controlled group across the central Pacific is of great importance to future global warming related studies and species distribution models. It may serve as an indicator of warming habitats as it establishes itself outside of its traditional geographical limits.

Niue and Beveridge Reef, like most coral reef islands, have a self-sustaining mechanism that maintains the islands through the deposition of calcium carbonate by marine organisms. Foraminifera are one of the dominant sand-grade sediment sources in Niue, and the implications of reef ecosystem change on stability and maintenance of this environment are profound. The sediments are produced on the platforms close to the beaches with little evident longshore transport, so the beaches are highly vulnerable to any change in either energy conditions or ecological disturbance affecting sediment supply. Failing to sufficiently monitor and manage the health of this unique ecosystem might result, amongst other outcomes, in the disappearance of beaches, and the very source of ‘land’ for some of the world's most at risk coral-atoll island nations.

## Data Availability

The datasets supporting this article have been uploaded as part of the electronic supplementary material [90].
